# High Prevalence and Partner Correlates of Physical and Sexual Violence by Intimate Partners among Street and Off-Street Sex Workers

**DOI:** 10.1371/journal.pone.0102129

**Published:** 2014-07-10

**Authors:** Elena Argento, Katherine A. Muldoon, Putu Duff, Annick Simo, Kathleen N. Deering, Kate Shannon

**Affiliations:** 1 Gender and Sexual Health Initiative, British Columbia Centre for Excellence in HIV/AIDS, Vancouver, British Columbia, Canada; 2 School of Population and Public Health, University of British Columbia, Vancouver, British Columbia, Canada; 3 Department of Medicine, University of British Columbia, Vancouver, British Columbia, Canada; University of Washington, United States of America

## Abstract

**Objectives:**

Intimate partner violence (IPV) is associated with increased risk of HIV among women globally. There is limited evidence and understanding about IPV and potential HIV risk pathways among sex workers (SWs). This study aims to longitudinally evaluate prevalence and correlates of IPV among street and off-street SWs over two-years follow-up.

**Methods:**

Longitudinal data were drawn from an open prospective cohort, AESHA (An Evaluation of Sex Workers Health Access) in Metro Vancouver, Canada (2010–2012). Prevalence of physical and sexual IPV was measured using the WHO standardized IPV scale (version 9.9). Bivariate and multivariable logistic regression using Generalized Estimating Equations (GEE) were used to examine interpersonal and structural correlates of IPV over two years.

**Results:**

At baseline**,** 387 SWs had a male, intimate sexual partner and were eligible for this analysis. One-fifth (n = 83, 21.5%) experienced recent physical/sexual IPV at baseline and 26.2% over two-years follow-up. In multivariable GEE analysis, factors independently correlated with physical/sexual IPV in the last six months include: childhood (<18 years) sexual/physical abuse (adjusted odds ratio [AOR] = 2.05, 95% confidence interval [CI]: 1.14–3.69), inconsistent condom use for vaginal and/or anal sex with intimate partner (AOR = 1.84, 95% CI: 1.07–3.16), <daily prescription opioid use (AOR = 1.72, 95% CI: 1.02–2.89), providing financial support to intimate partner (AOR = 1.65, 95% CI: 1.05–2.59), and sourcing drugs from intimate partner (AOR = 1.62, 95% CI: 1.02–2.26).

**Discussion:**

Our results demonstrate that over one-fifth of SWs in Vancouver report physical/sexual IPV in the last six months. The socio-structural correlates of IPV uncovered here highlight potential HIV risk pathways through SWs’ intimate, non-commercial partner relationships. The high prevalence of IPV among SWs is a critical public health concern and underscores the need for integrated violence and HIV prevention and intervention strategies tailored to this key population.

## Introduction

Male-perpetrated intimate partner violence (IPV) is a pervasive human rights violation and public health concern, with substantial negative impacts on morbidity and mortality, including poor sexual and reproductive health outcomes, HIV, and sexually transmitted infections (STIs) [1], [2]. It is estimated that up to 60% of women globally will experience physical and/or sexual violence in their lifetime, most commonly from their intimate partners: 30% of women worldwide who have ever been in a relationship have experienced physical and/or sexual IPV [3], [4]. IPV includes violence in the form of “sexually, psychologically and physically coercive acts used against adult and adolescent women by a current or former intimate partner, without her consent” [4].

Immediate consequences of IPV include injuries and death from physical assault, unintended pregnancies, HIV/STIs, and psychological distress [5]. Long-term conditions associated with IPV include chronic pain conditions, gastro-intestinal syndromes and other physical disabilities [6], post-traumatic stress disorder, depression, anxiety, substance abuse, and suicide [7], [8]. There are likely multifactorial pathways through which IPV increases risk for these adverse health outcomes and the direct effects of physical trauma and the long-term accumulation of stress may be key contributing factors [9]. The UN has declared an urgent need to strengthen the knowledge base on all forms of violence against women to inform policy and strategy development [10].

In North America, male-perpetrated IPV is associated with a significant burden. In the U.S., the 2010 National Intimate Partner and Sexual Violence Survey indicated that 30% of women experience physical IPV and 17% sexual IPV in their lifetimes [11]. In a 2003 review and critique of 16 Canadian prevalence studies, the annual prevalence of physical abuse among Canadian women ranged from 0.4% to 18%, and lifetime prevalence of physical or sexual abuse by their male partners ranged from 8.0% to 36.4% [12]. Another Canadian study that examined data from the 1999 Canadian General Social Survey for gender patterns of IPV found that being younger, divorced/separated or single, having children in the household, and poor self-rated health were significant risk factors for physical/sexual IPV [13]. The 2005 report on the WHO Multi-Country Study on Women’s Health and Domestic Violence Against Women estimated the lifetime prevalence of physical or sexual IPV among ever-partnered women to range from 15% to 71%, with past year prevalence estimated between 4% and 54% [14].

Other studies from around the world have documented the association between partner violence and gender inequality with increased risk of HIV [1], [15]–[17]. A 2010 longitudinal study of 1099 women (aged 15–26) from South Africa demonstrated strong temporal evidence between IPV and incident HIV infection: approximately one in seven incident HIV infections among the young women were attributable to either IPV or low gender equity in their relationships [18]. A 2013 longitudinal study in Uganda also found an association between lifetime IPV and risk of incident HIV infection, which tended to be greater for women who were exposed to more severe and frequent IPV and for a longer duration [19].

Despite growing data on the magnitude and correlates of IPV among the general population of women of reproductive age globally [20]–[22], there is a surprising dearth of research on IPV experiences among marginalized and stigmatized populations, such as sex workers (SWs), women who use drugs, homeless women and female youth, particularly in high income settings. Globally, SWs continue to face a disproportionate amount of violence [23]–[25]. While IPV among women who use drugs has received some attention [26]–[28], there are very few epidemiological studies on IPV among SW populations, and the majority of research has been done in lower-middle income countries (LMIC) such as India, Mexico, Kenya, and other Sub-Saharan African (SSA) settings [29]–[31]. Based on a recent global systematic review, past year physical/sexual IPV prevalence rates among SWs were estimated to range from 8% to 61%, while lifetime prevalence of any type of IPV (physical, sexual or emotional) ranged from 4% to 73% [25]. A longitudinal study in the U.S. of 416 women enrolled in methadone maintenance treatment programs found significant bi-directional temporal relationships between sexual and/or physical IPV and risk of sexual HIV/STI transmission (i.e. inconsistent/no condom use and IPV and inconsistent/no requests for partners to use condoms and IPV) [26].

Qualitative and ethnographic research among marginalized groups of women (street-involved SWs and young, homeless drug users) has documented the pervasiveness of controlling and abusive boyfriends, providing some contextual understanding around the power imbalances and associated violence that directly influences women’s agency and ability to safeguard against risky sexual and drug-using behaviors, making these populations particularly vulnerable to transmission of HIV/STIs [27], [28], [32]. As sexual exclusivity is highly valued in intimate relationships in Western societies, SWs and their intimate partners may struggle with notions of infidelity and trust within the context of sex work [33]. Other qualitative studies have suggested that intimate partners of SW may be jealous of clients [34], facilitating pathways to violence.

There is a critical need for research on IPV among marginalized groups, including SWs. The objectives of this study were therefore to examine the prevalence of physical and sexual IPV against a cohort of SWs in Vancouver, Canada and to describe the socio-structural correlates of IPV.

## Methods

### Study Design and Sample

Data for this study were drawn from AESHA (An Evaluation of Sex Workers Health Access), an open prospective cohort of female SWs (2010–2012) who conduct sex work in both street (public) and off-street (indoor) settings. Eligibility criteria for AESHA participants at baseline includes being female (including transgender, male-to-female), older than 14 years of age, having exchanged sex for money within the last 30 days, and providing written informed consent. This analysis is restricted to AESHA participants who reported having at least one intimate partner, which is defined as having a sexual, non-commercial, male partner in the last six months, at baseline.

In the context of hard-to-reach populations, SWs were recruited through time-location sampling and community mapping strategies. Day and late night peer-outreach was used to identify both outdoor sex work locations (i.e. streets, alleys) and indoor sex work venues (i.e. massage parlors, micro-brothels, and in-call locations) across Metro Vancouver. In addition, online recruitment was used to reach SWs working through online solicitations spaces.

At enrolment and on a bi-annual basis, participants complete an interview-administered questionnaire by a trained interviewer and HIV/STI/HCV (hepatitis C virus) serology testing by a project nurse. The main interview questionnaire elicits responses related to socio-demographics (e.g. sexual identity, ethnicity, housing), sex industry work (e.g. work environment, solicitation, social cohesion and support, access to services, violence and safety, incarceration), clients (e.g. number/type of clients, types of services, condom use), intimate partners (e.g. sexual history, cohabitation, financial support), trauma and violence (e.g. lifetime and childhood trauma, exposure to intimate partner and occupational violence), and drug use patterns (injection and non-injection). In addition, a clinical questionnaire is administered relating to overall physical, mental and emotional health, sexual and reproductive health, and HIV testing and treatment experiences. SWs have the option to visit one of two study offices or complete the questionnaire and nursing component at a safe location identified by them, including work or home locations. All participants receive an honorarium of $40CAD at each bi-annual visit for their time, expertise and travel.

### Ethics Statement

The AESHA study holds ethical approval through Providence Health Care/University of British Columbia Research Ethics Board and has a community advisory board of over 15 agencies.

### Study Variables

#### IPV Outcome

Recent IPV was measured using an abridged version of the WHO Standardized IPV Scale Version 9.9 [14], [35]. The scale was originally developed for the WHO Multi-country Study on Women’s Health and Domestic Violence Against Women in response to large discrepancies in research design and methods, making comparison of data between settings difficult. The standardized scale elicits responses from women about experiences of physically and sexually violent acts by a current or former intimate male partner, and about selected symptoms associated with physical and mental health. The three violence components (physical, sexual and emotional) are each validated separately and as a single scale [14], [35], [36]. For the purposes of this analysis, and due to substantial overlap between sexual and physical IPV, our outcome measure was moderate or severe physical and/or sexual violence by any male intimate (non-commercial) partner in the last six months and was time-updated at each follow-up visit. The emotional violence component of the WHO scale was not included in this analysis. Physical violence included both “moderate” (“yes” response to one or more of: slapped or thrown something; pushed or shoved) and/or “severe” (“yes” response to one or more of: hit with a fist; kicked, dragged or beaten up; choked or burnt; threatened to use or used a gun or other weapon), while sexual IPV included “yes” responses to one or more of the following: forced to have sex against will, having sex out of fear, and forced to perform degrading or humiliating sexual acts.

#### Individual and Socio-Demographic Variables

Study variables for potential correlates of IPV were selected based on the literature and available data collected for the AESHA cohort between 2010 and 2012. Fixed variables considered at baseline included demographic variables such as: age (continuous), sexual minority (lesbian, gay, bisexual, transgender, transsexual, or two-spirited), being of Aboriginal/Indigenous ancestry (inclusive of First Nations, Metis, and Inuit), and being a migrant/new immigrant worker (versus Canadian born). Historical exposure to childhood physical and/or sexual abuse (<18 years of age) was also included. Individual variables including frequency of use of injection and non-injection illicit drugs (daily, less than daily or no use) were time-updated, and based on the last six months at each follow-up.

#### Partner-Level Data

The study participants provided all information relating to their partners, as the partners themselves were not interviewed. Partner-level data were time-updated, co-variates were collected at baseline and each follow-up visit for the primary intimate sex partner, and included inconsistent condom use for vaginal/anal sex with intimate partners, condom refusal by intimate partner, cohabitating with intimate partner, sourcing drugs (not including pot or alcohol) from intimate partner, and financial support provided to or by an intimate partner. Whether or not intimate partners had other sexual partners (both commercial and non-commercial) was also included.

### Statistical Analyses

Analyses were restricted to AESHA participants who reported having at least one recent intimate (non-commercial) male sex partner (last six months). Socio-demographic variables (age, ethnicity, sexual minority, migrant status) were considered fixed variables. All other variables were considered time varying, and were updated to reflect their occurrence within the last six months. All time-updated variables were measured at the same time period as the outcome. Correlates of IPV were examined using bivariate and multivariable logistic regression using Generalized Estimating Equations (GEE), with a logit link for dichotomous variables. To adjust the standard error and account for correlations arising from the four repeated measurements on the same participant over the two-years follow-up period, an exchangeable correlation matrix was used. GEE accounts for missing data using the GEE estimating equation, that substitutes data from non-missing pairs into the estimators of the correlations matrix. Variables significantly associated with IPV at the p<0.05 level in bivariate screening were subsequently fitted into a multivariable GEE model to adjust for potential confounding. The multivariable model was constructed using Quasi-likelihood Information Criteria (QIC) selection, which has been used successfully in past research by our group [37]. The backward model selection procedure (QIC) identifies the model with the best overall fit as indicated by the lowest quasi-likelihood under the independence model criterion value [38]. Two-sided p-values and unadjusted and adjusted odds ratios (OR and AOR) with 95% confidence intervals (95%CI) are reported. All statistical analyses were performed using SAS software package version 9.3 (SAS Institute, Cary, NC, USA).

## Results

### Socio-Demographic Characteristics

Of the total cohort (n = 652), our analyses were restricted to participants who reported having at least one male, intimate sexual partner in the past six months for a sample of 387 street and off-street SWs. At baseline, one-fifth (n = 83, 21.5%) of women reported experiencing moderate or severe physical and/or sexual IPV in the last six months. The median age of all participants was 34 (interquartile range [IQR] = 28–41, minimum age = 17, maximum age = 58), with those who reported recent IPV being slightly younger than those who did not: 32 (IQR: 25–39) vs. 35 (IQR: 28–42) (p = 0.003). Most women (76.2%) were Canadian-born, and 39.0% self-identified as being of Aboriginal ancestry. Almost one quarter (24.3%) of participants reported being a sexual minority. The majority (66.7%) of SWs reported physical and/or sexual abuse before age 18 and this was higher among those who had experienced recent IPV compared to those who had not (84.3% vs. 61.8%) (p<0.001). Baseline socio-demographic and partner-level characteristics of participants who experienced IPV in the last six months compared to those who did not are displayed in Table 1.

**Table 1:** Socio-demographic and partner-level characteristics of sex workers (SWs) in Metro Vancouver who experienced physical and/or sexual intimate partner violence (IPV) compared to those who did not, at baseline.

Characteristics	Total N = 387	IPV n = 83	No IPV n = 304	*p*-value
**Sociodemographic Variables ** ***(n,%)***				
Age *(med, IQR)*	34 (28–41)	32 (25–39)	35 (28–42)	0.003
Canadian-born	295 (76.2)	73 (88.0)	222 (73.0)	0.003
Aboriginal ancestry	151 (39.0)	35 (42.2)	116 (38.2)	0.508
Physically or sexually abused before age 18	258 (66.7)	70 (84.3)	188 (61.8)	<0.001
HIV seropositivity	38 (9.8)	4 (4.8)	34 (11.1)	0.063
STI seropositivity	38 (9.8)	6 (7.2)	32 (10.5)	0.355
Sexual minority	94 (24.3)	20 (24.1)	74 (24.3)	0.963
Coerced into sex work	47 (12.1)	11 (13.3)	36 (11.8)	0.730
Daily prescription opioid use†	11 (2.8)	1 (1.2)	10 (3.3)	0.003
Less than daily prescription opioid use†	63 (16.3)	24 (28.9)	39 (12.8)	0.003
Injection drug use†	158 (40.8)	39 (47.0)	119 (39.1)	0.200
Non-injection drug use†	279 (72.1)	73 (88.0)	206 (67.8)	<0.001
**Partner-Level Variables ** ***(n,%)***				
Intimate Partner (IP) used injection drugs†	82 (21.2)	30 (36.1)	52 (17.1)	<0.001
IP used non-injection drugs†	246 (63.6)	67 (80.7)	179 (58.9)	<0.001
Inconsistent condom use in vaginal/anal sex with IP†	273 (70.5)	66 (79.5)	207 (68.1)	0.038
Condom refusal by IP†	10 (2.5)	5 (6.0)	5 (1.6)	0.033
Scored drugs from intimate partner†	145 (37.5)	49 (59.0)	96 (31.6)	<0.001
Financial support provided to IP†	122 (31.5)	39 (47.0)	83 (27.3)	<0.001
Financial support provided by IP†	241 (62.3)	46 (55.4)	195 (64.1)	0.149
IP has other SW sex partners†	48 (12.4)	12 (14.5)	36 (11.8)	0.528
IP has other non-SW sex partners†	53 (13.7)	16 (19.3)	37 (12.17)	0.107
Cohabitating with IP†	151 (39.0)	33 (39.8)	118 (38.8)	0.876

† In the last 6 months.

Note: Study participants provided all partner-level data.

Regarding drug use, 72.1% of SWs at baseline had used non-injection illicit drugs and 40.8% had injected drugs in the last six months. At baseline, the number of SWs who reported using prescription opioids (POs) less than daily was 63 (16.3%), which was higher among those with IPV (28.9%) than those without (12.8%) (p = 0.003). Non-injection and injection drug use by intimate partners was reported at 63.6% and 21.2%, respectively, and 37.5% of participants reported sourcing drugs from their intimate partners. In the last six months, 39.0% of SWs were living with their intimate partners and 13.7% of intimate partners had other sex partners.

### Bivariate & Multivariable GEE Analyses

Bivariate and multivariable odds ratios for correlates with recent IPV are displayed in Table 2. In the bivariate GEE analysis, factors found to be significantly positively correlated with recent physical/sexual IPV at a p<0.05 level included condom refusal for vaginal and/or anal sex by an intimate partner (Odds Ratio [OR] 4.48, 95% Confidence Interval [95%CI] 1.63–12.28), being Canadian-born (OR 3.36, 95%CI 1.69–6.69), childhood physical and/or sexual abuse <18 years (OR 3.34, 95%CI 1.89–5.90), non-injection drug use (OR 3.12, 95%CI 1.72–5.62), sourcing drugs from an intimate partner (OR 2.77, 95%CI 1.85–4.14), non-injection drug use by intimate partner (OR 2.61, 95%CI 1.63–4.18), injection drug use by intimate partner (OR 2.56, 95%CI 1.66–3.94), providing financial support to an intimate partner (OR 2.40, 95%CI 1.60–3.59), less than daily PO use (OR 2.38, 95%CI 1.46–3.88), inconsistent condom use in vaginal and/or anal sex with an intimate partner (OR 2.27, 95%CI 1.39–3.71), intimate partner had other non-SW sex partners (OR 2.03, 95%CI 1.08–3.80), and injection drug use (OR 1.66, 95%CI 1.12–2.47).

**Table 2:** Longitudinal bivariate and multivariate GEE of correlates of physical and/or sexual intimate partner violence (IPV) among street and off-street sex workers (SWs) with a male, intimate partner (IP) in the AESHA Cohort (n = 387).

	Unadjusted	Adjusted
Characteristic	Odds Ratio (95% CI)	Odds Ratio (95% CI)
Younger Age	0.95 (0.93–0.98)**	0.96 (0.93–0.98)**
Canadian-born (vs. Migrant)	3.36 (1.69–6.69)**	-
Physically and/or sexually abused before age 18	3.34 (1.89–5.90)**	2.05 (1.14–3.69)*
Daily prescription opioid use†	0.44 (0.07–2.82)	0.35 (0.05–2.62)
Less than daily prescription opioid use†	2.38 (1.46–3.88)**	1.72 (1.02–2.89)*
Inconsistent condom use in vaginal/anal sex with IP†	2.27 (1.39–3.71)**	1.84 (1.07–3.16)*
Condom refusal by IP†	4.48 (1.63–12.28)**	-
Financial support provided to IP†	2.40 (1.60–3.59)**	1.65 (1.05–2.59)*
Sources drugs from IP†	2.77 (1.85–4.14)**	1.62 (1.02–2.56)*
Non-injection drug use†	3.12 (1.72–5.62)**	1.96 (0.96–4.00)
Injection drug use†	1.66 (1.12–2.47)*	-
IP used non-injection drugs†	2.61 (1.63–4.18)**	-
IP used injection drugs†	2.56 (1.66–3.94)**	-
IP had other non-SW sex partners†	2.03 (1.08–3.80)*	-

*p<0.05.

**p<0.01.

† In the last 6 months.

Note: Study participants provided all partner-level data.

In the multivariable GEE analysis, factors independently correlated with recent physical/sexual IPV over the last six months include: childhood physical and/or sexual abuse <18 years (Adjusted Odds Ratio [AOR] 2.05, 95%CI 1.14–3.69), inconsistent condom use for vaginal and/or anal sex with intimate partner (AOR 1.84, 95%CI: 1.07–3.16), less than daily PO use (AOR 1.72, 95%CI 1.02–2.89), providing financial support to intimate partner (AOR 1.65, 95%CI 1.05–2.59), sourcing drugs from intimate partner (AOR 1.62, 95%CI 1.02–2.26) and younger age (AOR 0.96 per increasing year of age, 95%CI 0.93–0.98).

## Discussion

Our longitudinal study demonstrates that over one-fifth of SWs in Metro Vancouver report moderate or severe physical and/or sexual IPV in the last six months. Experiencing recent IPV was independently associated with early childhood exposure to physical and/or sexual abuse, while partner-level factors emerged as key correlates over the course of follow-up, including inconsistent condom use, economic dependence of male intimate partner on sex worker, and sourcing drugs from an intimate partner.

The high prevalence of recent IPV among street and off-street SWs in our study is a critical and neglected human rights and public health concern and underscores the pressing need to focus on marginalized and hidden populations. Our results support existing literature documenting elevated levels of violence faced by SWs in Vancouver [32], [39]–[41] and highlight important socio-structural factors that intersect with violence within SWs’ intimate, non-commercial partner relationships. While growing research has examined workplace violence (e.g., by clients, police, community members) against SWs [42]–[44], there are very few population-based studies that investigate the factors influencing violence within SWs’ intimate partner relationships. This is despite observations that SWs experience structural and individual factors that heighten their risk for IPV, including high rates of homelessness [45] high rates of childhood maltreatment [46], [47], and high rates of unplanned pregnancy [48], [49].

The overlap between gender inequality and heightened risk of HIV plays an important role in the context of IPV against SWs. It is estimated that SWs have more than 13-times increased odds of having HIV compared to the general female population [50], and physical and/or sexual IPV has been found to be significantly associated with both higher levels of HIV risk behaviors and incident HIV infection among women globally [1], [16], [18]. Research demonstrates that experiences of IPV are often an extension of unequal gender roles and power imbalances in relationships; higher gender inequity has been found to be independently associated with increased male-controlled sexual decision making power, perpetration of rape, unprotected sex and multiple/concurrent sex partners [15].

Among drug-using women in particular, power dynamics with their intimate partners often favour traditional gender roles where men exert significant control over the relationship, including negotiating sexual risk-reduction behaviours. Bi-directional temporal relationships between sexual and physical IPV and risk of HIV/STI transmission have been demonstrated among drug-using women in the U.S, where inconsistent/no condom use and requesting partners to use condoms was significantly associated with IPV [26].

Qualitative research among substance-using women in survival sex work underscores the role of structural violence and gendered power inequities in shaping HIV and the need to facilitate enabling environments [32]. The normalization of physical, sexual and emotional violence among drug-using women in street environments makes these populations particularly vulnerable, especially where economic dependence and drug sharing occurs within sexual partnerships [28], [32]. Furthermore, in the context of increasing misuse of POs and associated harms in North America, and in light of our study’s results correlating IPV with the use of POs, there is a need to further investigate the mechanism linking POs and IPV among SWs. Evidence suggests that the misuse of POs now constitutes the third highest level of substance use burden of disease in Canada, after alcohol and tobacco [51].

This study’s findings that childhood abuse is positively associated with recent IPV, often referred to as “re-victimization”, is consistently documented in many settings [7], including psychiatric populations [52], the general population [53], [54], and vulnerable populations such as injection drug users [55]. Experiences of violence in childhood tend to “normalize” the abuse, increasing the likelihood of re-victimization and perpetuating the cycle of violence. Meta-analyses have concluded that between 15%–79% of women with histories of childhood trauma experience sexual violence as adults [56]. Within this study, an alarming 67% of the sample reported experiencing physical or sexual abuse before the age of 18, reinforcing a cycle of violence that contributed to 3.34 times the odds of experiencing recent IPV (95% CI: 1.89–5.90). A comparable study of 300 female SWs in two Mexico-U.S. border cities found that those who experienced abuse as a child were also more likely (OR = 2.49, 95% CI: 1.52–4.10) to have experienced recent IPV [30]. Antecedent studies have also shown that among a cohort of street involved youth in Vancouver, moderate to severe childhood trauma scores were associated with entry into sex work [47]. These findings substantiate the need for structural interventions that increase child protections and prevent future violence and risk.

Although violence between partners occurs at the interpersonal-level, the larger macro-level context plays an important role in sustaining cultures of complacency that tolerate gender-based violence, including against SWs. The criminalized nature of the sex industry in Canada drives a culture of stigma among SWs that leads to a cycle of violence that is ultimately fuelled by power inequity. Laws that further marginalize SWs not only constrain their choices occupationally, but also undermine their health in general: stigma associated with sex work prevents SWs from accessing health care services needed for violence treatment and prevention [57].

Implementing screening instruments for IPV in reproductive/primary health care and low-threshold support settings for marginalized populations, may help to more accurately detect IPV and direct focus toward SWs’ often overlooked non-commercial relationships. However, there continues to be debate around the extent to which screening effectively improves health outcomes for women [58], [59]. New WHO practice and policy guidelines now discourage universal screening in the general population, based on a lack of evidence demonstrating that screening for IPV produces better outcomes for women [60], [61]. Among marginalized populations, the challenges with effective IPV screening remain related to implementation, follow-up and support, with new research suggesting value in a systems approach to IPV screening among key populations [62]. Stigma remains a primary barrier to accessing violence prevention and health care services for SWs. Thus, health care facilities and programs must adapt to improve access to this highly stigmatized group by providing sensitivity training and fostering environments free from discrimination at all levels [57], in partnership with sex work communities.

### Limitations

This study has a number of strengths and limitations that should be considered in the interpretation of our study. The longitudinal design and analytic methods (GEE) are considered strengths of this study, increasing the number of observations, and allowing for average estimates of the correlates of IPV over a two-year period. However, as our analyses did not allow for temporal associations, we were unable to determine causality between the study variables and IPV. Many of the variables examined in our study were sensitive (i.e. childhood abuse, drug use) and IPV is a highly stigmatizing topic, which may have resulted in under-reporting or respondent-driven reporting biases in violence by our participants. However, the WHO Standardized IPV Scale used in this study was designed to ask a limited number of questions pertaining to common acts in violent partnerships rather than requiring respondents to identify themselves as abused – an approach that has been shown to encourage greater disclosure of violence [35]. The scale uses a relatively conservative definition of IPV and is thus more likely to underestimate rather than overestimate the true prevalence of violence. Furthermore, interviews were conducted in safe and comfortable spaces by experienced interviewers. The exclusion of the emotional violence component of the scale in this study may be seen as a limitation, as it may have biased the associations found by underestimating IPV as a whole. However, without the development of sound methodology for eliciting and measuring emotional violence experiences in relation to physical and sexual violence, it is difficult to ascertain if emotional violence should be conceptualized as a risk factor for physical/sexual IPV or rather a constituent element. Although our findings may not be fully generalizable to other SW populations and settings, our study population included SWs from a wide-ranging representation of sex work environments.

## Conclusion

The magnitude of physical and/or sexual IPV reported by SWs in our study demonstrates a critical need to focus on marginalized and stigmatized SW populations. SWs remain entrenched in a cycle of violence that often started in childhood and continues to impact their current intimate relationships. Our findings highlight key factors associated with IPV, including childhood exposure to physical or sexual violence, inconsistent condom use with intimate partners, economic dependence and sourcing drugs from an intimate partner, as well as PO use among SWs. The correlates of IPV uncovered here highlight important socio-structural factors that intersect with violence within SWs’ intimate, non-commercial partner relationships and underscore the need for further prevention and intervention strategies tailored to this key population, who continue to experience a disproportionate burden of violence.
